# Retraction of: Sequencing of Human Identification Markers in an Uyghur Population Using the MiSeq FGx^TM^ Forensic Genomics System

**DOI:** 10.1093/fsr/owaf039

**Published:** 2025-11-13

This is a retraction of: Halimureti Simayijiang, Niels Morling, Claus Børsting, Sequencing of Human Identification Markers in an Uyghur Population Using the MiSeq FGx™ Forensic Genomics System, *Forensic Sciences Research*, Volume 7, Issue 2, June 2022, Pages 154–162, https://doi.org/10.1080/20961790.2020.1779967

In September 2020, *Forensic Sciences Research* published the article ``Sequencing of Human Identification Markers in an Uyghur Population Using the MiSeq FGx™ Forensic Genomics System'' by Halimureti Simayijiang, Niels Morling, and Claus Børsting: https://doi.org/10.1080/20961790.2020.1779967

In late 2023, the editors and the publisher were alerted to concerns, including regarding the applicability of the ethical approval for the research, as noted in an Expression of Concern [1]. The `Compliance with ethical standard' section of the article read `According to the Danish Act on Research Ethics Review of Health Research Projects, the work did not require approval'. After an extensive investigation including the co-authors' institution and the relevant local ethical committee, the Publisher has been unable to ascertain definitively whether this was the case. Given this uncertainty, the journal is retracting the article and removing the supplementary files to the article. The authors have been informed of this decision and do not agree with the retraction.

[1] Expression of Concern: Sequencing of Human Identification Markers in an Uyghur Population Using the MiSeq FGx™ Forensic Genomics System. *Forensic Sciences Research*, Volume 9, Issue 1, March 2024, owae010, https://doi.org/10.1093/fsr/owae010

